# Residual foreign body in the neck after trauma results in the delayed rupture of the common carotid and internal jugular vein: a case report

**DOI:** 10.1186/1752-1947-7-13

**Published:** 2013-01-10

**Authors:** Yan Luo, Hui Yuan, Zhong Sheng Cao

**Affiliations:** 1 Department of Otorhinolaryngology, The Second Affiliated Hospital of Soochow University, Soochow, Jiangsu Province, China

**Keywords:** Common carotid, Foreign body, Internal jugular vein, Trauma

## Abstract

**Introduction:**

Trauma and foreign body residue occurring in different settings are common in the neck. Some small injuries go unrecognized, and vascular injuries caused by the sharp penetrating trauma of a foreign body are very dangerous. Without early diagnosis and treatment, foreign body residue remains a major cause of mortality.

**Case presentation:**

A six-cm piece of wooden chopstick was not initially detected in the neck of a 24-year-old Chinese man presenting with a slight bleeding wound after a brawl accident. Three days later, the patient had an expanding neck hematoma and shortness of breath. Computed tomography revealed a dense shadow in the soft tissue of the left side of the patient’s neck, and surgical exploration found that a residual broken chopstick had resulted in a delayed rupture of the common carotid artery and internal jugular vein.

**Conclusion:**

A residual foreign body should be seriously considered after neck trauma because it can result in a lethal hemorrhage originating from a delayed rupture of blood vessels.

## Introduction

Trauma and foreign body residue occurring in different settings are common in the neck. Some small injuries go unrecognized, and vascular injuries caused by the sharp penetrating trauma of a foreign body are very dangerous. Without early diagnosis and treatment, foreign body residue remains a major cause of mortality. Here we report an unusual case in which a chopstick stabbed into the patient’s neck initially resulted in subcutaneous emphysema and slight bleeding, but three days later a rupture of the common carotid artery (CCA) and internal jugular vein (IJV) caused hemorrhage in the neck after a sudden bout of coughing.

## Case presentation

A 24-year-old Chinese man was admitted to our Emergency Department presenting with a neck wound that had been slightly bleeding for six hours. The neck trauma occurred in a chaotic fight. The patient could not describe details of the injury, but he reported that the wound might have been caused by fragmentation of a beer bottle. On physical examination, there were no signs or symptoms of respiratory distress. The wound opening, measuring one cm, was identified at the submandibular area of the left side of the patient’s neck below the border of the mandibular angle. No glass fragments or other foreign bodies were detected, but slight swelling and bleeding were noted. Examinations of the oral cavity and pharynx revealed no abnormalities.

**Figure 1:**
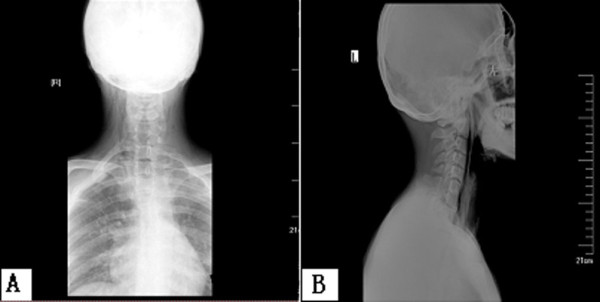
**The anteroposterior (a) and lateral view X-ray (b) of the neck.** It reveals subcutaneous emphysema but no foreign body was observed in the soft tissues.

**Figure 2:**
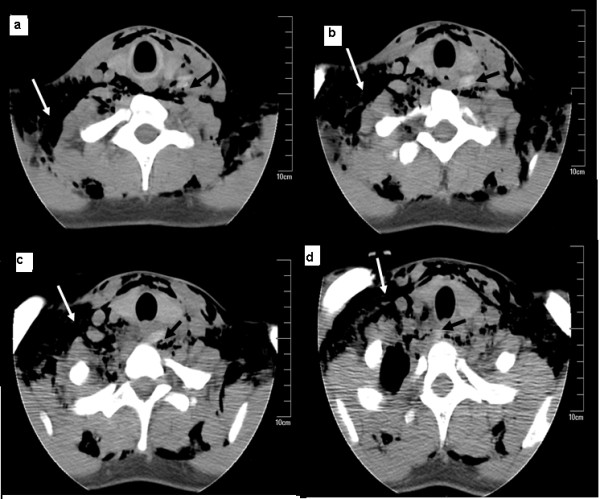
**Plain computed tomography scan of the neck (a,b,c,d) obtained on the third day after the accident.** The computed tomography scan shows extensive subcutaneous emphysema in the neck and upper breast (white arrowhead). A dense shadow is visualized as a linear structure (black arrowhead) at the level of the 7th cervical vertebra and 1st dorsal vertebra pointing towards the common carotid artery.

Anteroposterior and lateral X-rays of the patient’s neck revealed subcutaneous emphysema and no foreign body in the soft tissues (Figure 1). The wound was closed by suturing. The patient was then given antibiotics and referred to the Department of Stomatology ward for observation. There was significant relief of the subcutaneous emphysema and wound swelling in the first two days. However, he still felt pain and the movement of his neck was restricted. On the third day after a bout of violent coughing he had an abrupt onset of left-sided neck swelling with neck pain and shortness of breath. Physical examination discovered that the trachea was deviated to the right without any neurological deficit. A computed tomography (CT) scan revealed extensive subcutaneous emphysema in the neck and upper breast region, and a dense linear shadow at the level of the 7th cervical vertebra and 1st dorsal vertebra whose ends were embedded in the pre-vertebral soft tissue and parapharyngeal space (Figure 2).

A tracheotomy was promptly performed to relieve his shortness of breath. Once the skin and subcutaneous tissue were opened, a massive amount of fresh blood and blood clots poured out of the incision. Our initial clinical impression was that he had ruptured cervical arteries, and a cervical hematoma resulted in compression and marked displacement of the airway to the right. As the ongoing bleeding could not be controlled, an emergency exploration of the patient’s neck was initiated while an assistant compressed the bleeding site with gauze. His neck was opened on the left side under general anesthesia. On opening his platysma and retracting the sternocleidomastoid muscle laterally, a rapidly expanding and bulging hematoma was observed extending superiorly into the parapharyngeal space and inferiorly into the superior mediastinum. After evacuating the hematoma, an arterial tear was identified at the posteromedial wall of the CCA. The IJV wall was also torn in the region adjacent to the tear of the CCA. The points of perforation were associated with arterial pulsatile postoperative bleeding and venous extravasation of blood components, with surrounding adventitia appearing somewhat ragged, suggesting a pricking injury. A broken chopstick was noted lying across the pre-vertebral soft tissue with its tip embedded in the CCA and its butt in the right cervical pleura. The six-cm piece of chopstick was removed. The vascular defects were repaired with polypropylene 6–0 suture at the injury site, located approximately two cm distal to the bifurcation.

**Figure 3:**
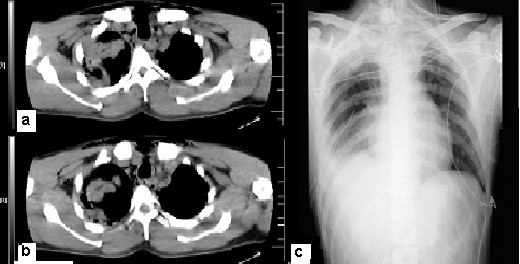
**Computed tomography scan (a,b) and the anteroposterior view X-ray (c) of the chest after cervical surgery.** It revealed a pneumothorax, much fluid, and pulmonary atelectasis on the right side of the chest with pneumomediastinum.

The patient was intubated and mechanically ventilated in the intensive care unit. A CT scan and an anterior-posterior view X-ray of the chest following cervical surgery revealed a pneumothorax, much fluid, and pulmonary atelectasis on the right side of the chest with pneumomediastinum (Figure 3). A chest tube and closed drainage system was used to remove the gas and fluid from the intrathoracic space. A total of 800mL of dark red bloody fluid was drained. The hemopneumothorax resolved by the third day after the operation, and the chest tube was taken off suction. Mechanical ventilation was discontinued postoperatively the same day. A further CT of the chest showed that encapsulated pleural effusion was present in the right upper and lower lobes. Video-assisted thoracoscopic surgery was performed for the debridement and deloculation of the clotted hemothorax. In all, 400mL of retained clotted blood was cleared with a suction instrument intraoperatively. The patient was discharged one month later.

## Discussion

Residual foreign bodies in the neck might result in life-threatening complications. This patient presented with a delayed rupture of the CCA and IJV, subcutaneous emphysema, pneumomediastinum, and hemopneumothorax. The residual foreign body might damage the tunica media with intact adventitia, and lead to the formation of a pseudoaneurysm. Presentation can be delayed for several days, and is usually with a hematoma and pressure symptoms because of minor bleeding [1]. Severe bleeding from arterial damage is catastrophic and is even fatal [1]. Moreover, the hematoma might compress and displace the trachea, causing airway obstruction or choking. In this case, the foreign body pierced the right cervical pleura, causing air to leak through the perforation into the mediastinum and pleural space.

Two underlying mechanisms might account for the delayed rupture of the CCA and IJV caused by this residual foreign body. The action of swallowing or violent coughing might have induced the migration of the sharp broken chopstick close to adjacent vessels, and its tip pricked into the vessel wall. In addition, the residual chopstick could have caused the formation of a pseudoaneurysm, which eventually led to the massive bleeding from the delayed rupture of the neck vessels.

The residual broken chopstick was not detected until continuous bleeding was manifest, with a delay of three days from injury to definitive diagnosis. This delay should be attributed to two facts. First, the patient did not provide a detailed history of the trauma. The possibility of a residual foreign body was not taken seriously because the mild stab wound only exhibited slight bleeding and no hard objects were palpated by physical inspection. Second, the doctor did not emphasize the occurrence of subcutaneous emphysema on the first visit, and CT scanning of the chest was not performed. As a result, no efforts were made to discover the reason for the hemopneumothorax that was caused by the neck trauma.

Detection of wooden foreign bodies is difficult from a plain X-ray. In this case, a plain X-ray of the neck did not reveal the foreign body impacted in soft tissue. This was probably due to the fact that there was similar transmitted light under X-ray between the wooden object and nearby soft tissue. A radiopaque foreign body might be obscured by the opacity of cervical vertebrae, resulting in the disappearance of the foreign body on a common radiograph. Kantarci *et al*. [2] reported that the entry of a wooden foreign body into the neck was detected neither in the emergency department nor during conventional radiograms. CT scanning has been demonstrated to be an effective imaging modality in detecting wooden foreign bodies. The cross-sectional view on CT imaging is beneficial in determining the location of the foreign body and its relationship to vital structures in the neck [3]. Sonography is a useful modality in detection and localization of radiolucent foreign bodies in soft tissue which can avoid misdiagnosis during primary emergency evaluation [4]. Jacobson *et al*. [5] demonstrated that ultrasound can effectively locate 2.5mm-long wooden foreign bodies with a sensitivity and specificity of 86.7% and 96.7%, respectively. The sensitivity and specificity of ultrasound for the detection of 5.0mm-long wooden foreign bodies has been demonstrated to be 93.3% and 96.7%, respectively [5]. The early detection of a residual foreign body eliminates the risk of the delayed rupture of a vessel.

## Conclusion

In conclusion, delayed rupture of the CCA is an uncommon yet potentially life-threatening condition, which can be caused by a residual foreign body following trauma. A physical examination and radiogram cannot exclude the possibility of a residual wooden foreign body, particularly when a clear history of trauma is unobtainable. CT scanning and ultrasound are extremely helpful for the early detection and extraction of such a foreign body.

## Consent

Written informed consent was obtained from the patient for publication of this case report and accompanying images. A copy of the written consent is available for review by the Editor-in-Chief of this journal.

## Abbreviations

CCA: Common carotid artery; CT: Computed tomography; IJV: Internal jugular vein.

## Competing interests

The authors declare that they have no competing interests.

## Authors’ contributions

YL collected the information and complete history of the patient and wrote the article. ZC interpreted the patient’s data regarding hematological disease and edited the manuscript. HY was the main supervisor and surgeon. All authors read and approved the final manuscript. All authors contributed equally to this case presentation.
